# Evolution of light-harvesting complex proteins from Chl *c*-containing algae

**DOI:** 10.1186/1471-2148-11-101

**Published:** 2011-04-15

**Authors:** Gabriel E Hoffman, M Virginia Sanchez-Puerta, Charles F Delwiche

**Affiliations:** 1 Department of Cell Biology and Molecular Genetics, University of Maryland, College Park, MD, 20742, USA; 2 Maryland Agricultural Experiment Station, University of Maryland, College Park, MD, 20742, USA; 3 Department of Biological Statistics and Computational Biology, Cornell University, Ithaca, NY, 14853, USA; 4 IBAM, Facultad de Ciencias Agrarias, Universidad Nacional de Cuyo, Chacras de Coria, 5500 Mendoza, Argentina

## Abstract

**Background:**

Light harvesting complex (LHC) proteins function in photosynthesis by binding chlorophyll (Chl) and carotenoid molecules that absorb light and transfer the energy to the reaction center Chl of the photosystem. Most research has focused on LHCs of plants and chlorophytes that bind Chl *a *and *b *and extensive work on these proteins has uncovered a diversity of biochemical functions, expression patterns and amino acid sequences. We focus here on a less-studied family of LHCs that typically bind Chl *a *and *c*, and that are widely distributed in Chl *c*-containing and other algae. Previous phylogenetic analyses of these proteins suggested that individual algal lineages possess proteins from one or two subfamilies, and that most subfamilies are characteristic of a particular algal lineage, but genome-scale datasets had revealed that some species have multiple different forms of the gene. Such observations also suggested that there might have been an important influence of endosymbiosis in the evolution of LHCs.

**Results:**

We reconstruct a phylogeny of LHCs from Chl *c*-containing algae and related lineages using data from recent sequencing projects to give ~10-fold larger taxon sampling than previous studies. The phylogeny indicates that individual taxa possess proteins from multiple LHC subfamilies and that several LHC subfamilies are found in distantly related algal lineages. This phylogenetic pattern implies functional differentiation of the gene families, a hypothesis that is consistent with data on gene expression, carotenoid binding and physical associations with other LHCs. In all probability LHCs have undergone a complex history of evolution of function, gene transfer, and lineage-specific diversification.

**Conclusion:**

The analysis provides a strikingly different picture of LHC diversity than previous analyses of LHC evolution. Individual algal lineages possess proteins from multiple LHC subfamilies. Evolutionary relationships showed support for the hypothesized origin of Chl *c *plastids. This work also allows recent experimental findings about molecular function to be understood in a broader phylogenetic context.

## Background

Light harvesting complex (LHC) proteins are fundamental to oxygenic photosynthesis, and members of the LHC family are present in most photosynthetic eukaryotes, although variation in nomenclature sometimes obscures their widespread occurrence (Table 1). These transmembrane proteins bind chlorophyll (Chl) and carotenoid pigments which function to absorb light and transfer energy to the reaction center Chl of photosystems (PS) in the thylakoid membrane [1]. The biochemistry, physical interactions and molecular phylogeny of multiple types of LHCs have been characterized in plants and chlorophytes (green algae) [2-4], but less is known about homologs in Chl *c*-containing algae. Consequently, to improve our understanding of the evolution of the LHC gene family in Chl *c*-containing lineages, we undertook a phylogenetic analysis of expressed sequence tags (ESTs) and genomic data. Chl *c*-containing algae, along with their non-photosynthetic relatives are also known as "chromalveolates" [5] under the hypothesis that these lineages descend from a photosynthetic common ancestor. However, the monophyly of the "chromalveolates" has been questioned and remains controversial [6,7]. This analysis is intended to relate the molecular phylogeny of the Chl *c*-containing algal LHCs to their function, and provides insight into gene duplication, expression, differential biochemical activity and evolution of the LHC family, and although we use the term here, the study is not dependent upon the validity of the chromalveolate hypothesis.

**Table 1:** Major clades of the LHC gene superfamily.

Systematic name	Other names/subclades	Included in this analysis	Lineages
Lhca	LHC I	-	plants

Lhcb	LHC II, CP24,CP26, CP29	-	plants

Lhcc	Cac	+	cryptophytes

Lhcd	Lhcp	+	peridinin-containing dinoflagellates

Lhcf	FCP, cac	+	haptophytes, heterokonts

Lhcr	LhcaR	+	rhodophytes

--	Lhcz	+	cryptophytes, haptophytes, heterokonts

--	LI818, LHCSR	+	chlorarachniophytes, chlorophytes, fucoxanthin-containing dinoflagellates, haptophytes, heterokonts

The systematic nomenclature was established by Jansson et al. [71]. The clades termed Lhcz and LI818 do not have systematic names and were identified by Koziol et al. [2] and Gagne and Guertin [72], respectively. None of the other clades in the current analysis have been previously described. The genes in Lhca, Lhcb and related clades recently identified by Koziol et al. [2] form a monophyletic outgroup to the genes analyzed in the current analysis, but the root of the phylogeny cannot be confidently inferred.

All eukaryotic lineages that are capable of oxygenic photosynthesis obtained this ability by (directly or indirectly) engulfing photosynthetic cyanobacteria and incorporating them as plastids [8], which are most familiar as the chloroplasts of green algae and plants. Chlorophytes, rhodophytes and glaucocystophytes appear to be the result of a single primary endosymbiotic event, whereby a cyanobacterium was incorporated as a plastid [9]. It should be noted that the 'event' in question could have involved many individual cells in a population, and might have resulted from processes that took place over a very long time, but all primary plastids seem to share a common origin, and hence it is appropriate to think of it as a single event [10,11]. Chl *c*-containing plastids are the result of at least one secondary endosymbiotic incorporation of a rhodophyte, and tertiary events involving a plastid of rhodophyte origin [5,7,8]. Chl *c*-containing algae include four major lineages: heterokonts, haptophytes, dinoflagellates and cryptophytes that are united (at least) by their use of Chl *c *as a photopigment. These lineages have often been treated separately, but recent evidence suggests that heterokonts and dinoflagellates (together with other alveolates) may form a single clade (possibly also including Rhizaria), and that cryptophytes and haptophytes are sister lineages [12,13]. The precise origin of the plastids (and number of endosymbiotic events involved) in these lineages is still unclear, but their ultimate rhodophyte ancestry is uncontroversial [14-16]. In addition, some dinoflagellates are thought to have replaced their plastids with new plastids from other lineages, including an environmentally important group (fucoxanthin-containing dinoflagellates) that have incorporated a haptophyte as a plastid in a tertiary endosymbiotic event [17]. Most recently (and controversially), genome analyses led Moustafa et al. [18] to hypothesize that the common ancestor of heterokonts and haptophytes acquired a transient green algal plastid, subsequently replaced by a red algal plastid and now only represented by residual genes of green algal origin.

Members of the LHC gene family are nuclear-encoded, and in organisms with secondary or tertiary plastids the proteins are targeted from the cytosol to the ER and then to the plastid using a bipartite signal sequence at the amino terminus. Functional LHCs are located in the thylakoid membrane of the plastid and possess three α-helical transmembrane regions (TMR) that are evolutionarily conserved [19]. The 3-dimensional structure and carotenoid binding sites of Chl *a*/*b *LHCs have been characterized [20,21], but the structural details and specific binding properties of the Chl *a/c *LHCs addressed here are likely different given the degree of sequence divergence and duration of independent evolution of these two gene subfamilies. Moreover, even closely related members of the diverse Chl *a/c *LHC family exhibit differential carotenoid and Chl binding, and differential associations in trimers or higher oligomers [22,23]. Thus, functional inferences made for plant LHCs can only tenuously be extended to the Chl *a/c *LHCs. However, a first step towards understanding these LHCs is to unravel evolutionary relationships within the gene family.

Major groups of algae can be characterized by the type of Chl associated with their LHCs. Chlorophyte and charophyte LHCs bind Chls *a *and *b*, rhodophyte LHCs bind Chl *a*, and Chl *c*-containing algae have LHCs that bind Chls *a *and *c*. These proteins also bind carotenoids that expand the absorption spectrum and are especially important in aquatic photosynthesis [24]. A range of carotenoids is present in algal plastids, with the relative abundance of each carotenoid varying between species. There is diversity in the most abundant carotenoid among these groups, with chlorophytes having lutein, rhodophytes zeaxanthin, and lineages of Chl *c*-containing algae have several distinct primary carotenoids. More specifically, cryptophytes have alloxanthin, most heterokonts and haptophytes have fucoxanthin, dinoflagellates with a plastid of red algal origin have peridinin (hereafter "peridinin-containing dinoflagellates"), while dinoflagellates with a plastid of haptophyte origin (hereafter "fucoxanthin-containing dinoflagellates") have a fucoxanthin derivative found in haptophytes [17,25]. It is generally thought that the primary carotenoid in each lineage constitutes the major component of LHC-bound pigments. Thus, LHCs naturally bind the Chl and carotenoid molecules present in their native lineage, but reconstruction experiments have shown that they are capable of binding non-native pigments [26]. Differences in primary pigment binding are at least as much a function of organismal biology as they are the result of fundamental differences in LHC biochemistry, although affinities for specific pigments do vary.

Most of the current biochemical, structural and expression research has focused on chlorophytes or plants and there are relatively few data on Chl *c*-containing algal LHCs. Previous biochemical studies of Chl *c*-containing algal LHCs have focused on *Cyclotella *spp., *Phaeodactylum tricornutum *and *Amphidinium carterae *[22,27,28]. Molecular sequences from a significant number of species have been published, but previous comparative or phylogenetic analyses have been limited to individual genomic sequences [e.g. [29]] or have not focused on Chl *a/c *sequences [e.g. [2]]. Presumably because of these limitations, some previous studies suggested that each Chl *c*-containing lineage possessed a single unique LHC subfamily [e.g. [29]]. Other studies identified a subfamily of LHCs that is highly conserved between chlorophytes and diatoms, and on that basis concluded that a single lineage possesses multiple subfamilies of LHCs and that the subfamily was not lineage specific [30,31]. However, no previous study has undertaken a comprehensive analysis of this complex gene family in Chl *c*-containing algae. Consequently, much current research on these LHCs is based on tenuously supported assumptions about relationships within the gene family. In addition, new biochemical [22,23,32] and expression data [32-36] on LHCs have recently become available that have not previously been related to the phylogeny of the protein family.

## Results and Discussion

### Overview of the Phylogeny

To help ensure that the gene phylogeny was comprehensive, but bounded by objective criteria, sequences were selected by BLAST analysis with a relatively low threshold, and then screened for the feasibility of end-to-end alignment. Analysis by motif detection programs indicated that the three TMRs are well conserved compared to the regions exposed to the plastid stroma and thylakoid lumen. These conserved regions generally included carotenoid and Chl binding sites. This protein architecture of three conserved TMRs was used to identify a set of LHCs that were practical for analysis.

**Figure 1:**
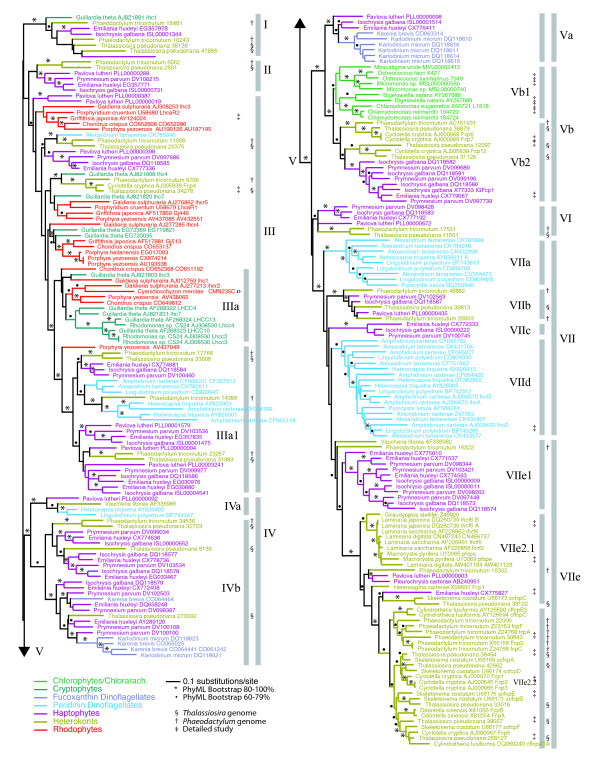
**The maximum likelihood tree from PhyML, with 246 taxa based on a 266 aa alignment (PhyML/AA)**. The individual LHCs are colored by taxonomic lineage: chlorophytes and chlorarachniophytes (green), cryptophytes (turquoise), fucoxanthin-containing dinoflagellates (light purple), peridinin-containing dinoflagellates (light blue), haptophytes (dark purple), heterokonts (brown), rhodophytes (red). The taxon name and sequence identifier are given for each sequence, and gene name is given when available. See Additional File 1, Additional Figure S1 for complete support values from other analyses.

Phylogenetic analyses were performed with a variety of methods and analytical conditions to ensure that the major features of the tree topology were not method-dependent. Analyses were performed using maximum likelihood methods implemented in PhyML [37] for an amino acid alignment and Garli [38] for the corresponding nucleotide alignment. Bayesian methods implemented in MrBayes [39] were used to analyze both alignments. In general, agreement was good among the analyses for features with strong bootstrap support (>80%) or posterior probability (>0.95), but substantial differences were observed in features with low support. Among the analyses performed (see *Materials and Methods *below), the tree found by PhyML with amino acid data (PhyML/AA) was a good representation of the overall analytical findings, and had relatively high support for features also observed in the other analyses (Figure 1). Particularly among deep branches, the PhyML/AA tree is well resolved compared to other analyses. The support values from the other analyses are given in Additional File1, Figure S1. To facilitate discussion of what is potentially a very confusing gene phylogeny, we have established a systematic nomenclature for well-supported clades (Figure 1). The tree is also labeled with corresponding protein names that are in most cases based on biochemical analyses in specific model systems, but because not all of the clades identified here have been characterized biochemically (or directly shown to be functionally distinct), we prefer a nomenclature that reflects the gene phylogeny because we feel it is likely to be relatively stable.

Our analysis is particularly reliable because we apply multiple robust statistical methods of tree reconstruction and include roughly 10-fold denser sampling of LHCs from Chl *c*-containing taxa and related species. Simulations have demonstrated that the probability-based methods employed here are superior in terms of reconstruction accuracy [40] compared to the methods used by Durnford et al. [29]. Moreover, Zwickl and Hillis [41] demonstrate that increased taxon sampling has a marked effect on reconstruction accuracy so that the phylogeny presented here is expected to be substantially more reliable for the relevant LHC proteins than that of Koziol et al. [2]. Distance optimality, neighbor-joining and parsimony based methods had lower support for most clades, but were compatible with the maximum likelihood phylogeny (data not shown).

The inferred phylogeny is unrooted (see below, in *Comparison to LHC superfamily*) and resolves the LHCs into seven major clades (I-VII), several of which are defined by a long, well-supported branch (Figure 1). The clades vary substantially in number of members and representation of algal lineages.

It is clear that most organismal lineages contain LHCs from multiple subfamilies in addition to duplicates with very similar sequences. This refutes the still widely accepted hypothesis that each LHC subfamily is characteristic of a particular group of organisms. Furthermore, there are many cases where clusters of genes represent several different organismal lineages, and some such clusters appear multiple times within the tree. The most common of these is the association of sequences from three Chl *c*-containing lineages (haptophytes, heterokonts and dinoflagellates). There are also some distinctive absences, for example, cryptophyte LHCs are only present in clades I and III, and rhodophyte LHCs are present only in clade III, while sequences from chlorophytes are present only in clade V. Taken together, these observations strongly imply that LHCs subfamilies are functionally distinct and under differential selection in many organisms.

### The role of endosymbiosis in the phylogeny

There is reason to think that the transfer of LHCs from endosymbiont to host genome has occurred repeatedly in the evolution of the gene family. The ancestral rhodophyte and chlorophyte plastid genomes were inherited from cyanobacterial ancestors, with relatively early and rapid transfer from the plastid to the host cell nuclear genome [42]. This transfer presumably occurred subsequent to the permanent incorporation of the plastid in the host cell, although this is not necessarily the case. Particularly in the context of kleptoplastidic organisms that appear to have obtained some genes from their prey [e.g. [43]], it is reasonable to think that some transfer of genes may have occurred prior to the acquisition of a plastid *per se *[10]. This is further complicated by the transfer of plastids via secondary and tertiary endosymbioses [8], followed by gene transfer from the eukaryotic endosymbiont to the host nuclear genome.

The overall structure of the phylogeny is consistent with the hypothesis that Chl *c*-containing algal LHCs descended from the rhodophyte proteins to create the diversity of extant sequences, although the high level of sequence divergence makes it difficult to infer the root. Related LHCs from chlorophytes (Lhca and Lhcb) constitute an outgroup, but have such low sequence similarity to those studied here that it was not possible to infer the root with reasonable confidence. Koziol et al. [2] and Durnford et al. [44] placed representatives of the set of proteins studied here (specifically clades V and VII) as sister to the group containing Lhca and Lhcb. Durnford et al. [29] placed the set as sister to Lhcb and some Lhca proteins with other Lhca proteins as an outgroup, although this arrangement did not have strong bootstrap support. The rooting shown here is compatible with those hypotheses, but several alternate rootings would be equally plausible.

The position of LHCs from the chlorarachniophyte *Bigelowiella natans *deeply nested among chlorophytes is expected because the *B. natans *plastid is of chlorophyte origin. Similarly, LHCs from fucoxanthin-containing dinoflagellates cluster with haptophytes, the source of their tertiary plastids [17]. If the diversification of the Chl *c*-containing algal LHCs entirely predated the acquisition of plastids from red algae then one would predict that each major clade in the gene phylogeny would have red algal sequences at its base. This is not observed, but it may be an artifact of incomplete data, as the only complete red algal genome analyzed here was from the highly reduced picoeukaryote *Cyanidioschyzon merolae*. We expect that other red algae will show a more diverse set of LHC genes.

### Diversity within individual lineages

Much of the diversity of LHC proteins - both within and among organisms - seen here (Figure 1) has not been previously characterized. Previous phylogenetic studies have included a relatively small number of LHC subfamilies, most of which were assumed to be lineage specific [29,44,45]. In addition, some algal lineages were thought to have multiple LHCs belonging to a single or a small number of LHC subfamilies. The current analysis greatly expands the number of putative subfamilies and indicates that some are lineage specific while others are not.

Previous studies [29,30,45-47] concluded that dinoflagellates possess only a single LHC subfamily (i.e. VIId), which was taken to be lineage specific. However, these analyses included only between 1 and 4 dinoflagellate sequences, all from *Amphidinium carterae*. Similarly, haptophytes were thought to possess LHCs from only a single subfamily that grouped with diatom and chlorophyte sequences in subfamily Vb [29,30,46]. A haptophyte LHC was later identified in clade VII [47]. A single large lineage specific gene subfamily was identified in heterokonts in the above analyses, corresponding to the clade VIIe2 of heterokonts in this study. Eppard et al. [30], Green [47] and Koziol et al. [2] incorporated more sequence data and identified heterokont sequences that grouped with rhodophytes in clade III and chlorophytes in clade V.

The incorporation of newly available sequence data illustrates the diversity and complexity of LHCs. In clade VII, which constitutes the major Chl *c*-containing clade, heterokonts contain a lineage specific LHC subfamily with sequences from diatoms and brown algae (VIIe2). Similarly, peridinin dinoflagellates possess LHCs from the lineage specific subfamilies VIIa and VIId. Subfamilies specific to haptophytes are VIIc and VIIe1, but in other clades sequences from this lineage often have close homologs in diatoms (e.g. VI and VIIb). Clade V comprises subfamilies from haptophytes, diatoms, fucoxanthin-containing dinoflagellates, chlorophytes and chlorarachniophytes. Clade III includes sequences from many lineages, and has subfamilies that are unresolved or poorly supported. In this case, cryptophyte and rhodophyte LHCs group together, as do those from diatoms, dinoflagellates and haptophytes.

### LHCs from complete genomes and possible biases in the data

At the time of this study, the only relevant complete genomes with substantial representation of the LHC family considered in this analysis were the diatoms (heterokont) *Thalassiosira pseudonana *[48] and *Phaeodactylum tricornutum *[49] (Additional File 2, Additional Table S1). Our analysis of *T. pseudonana *and *P. tricornutum *identified members of all seven LHC subfamilies, representing nearly the full diversity of sequences studied (Figure 1). The LHCs of these species are the only representatives from diatoms in most subfamilies outside of VIIe2, (with the exception of those from *Cy. cryptica)*. This is an important indication that other techniques are likely to miss members of the family. Indeed, EST and other transcriptomic studies almost always represent a subset of all of the genes present within the genome. Furthermore, with the exception of those from complete genome analyses (Additional File 2, Additional Table S1), the individual genomic sequences included in the analysis were obtained via Western blots using an antibody from a similar LHC [e.g. [50]], Southern blots using a probe from a similar LHC [e.g. [51]] or PCR using primers based on an LHC alignment [e.g. [52]]. Because these methods only detect proteins that are similar to the LHC used to create the antibody, probe, or primers, the set of sequences identified by these methods is intrinsically limited, and consequently this creates another source of sampling bias.

Taken together, these observations indicated that the diversity in LHCs reported here is an underestimate. It is also noteworthy that only a small fraction of the sequences included in this analysis have been characterized biochemically. Because sequence annotation often relies on the assumption that homologous sequences share biochemical function, a complex gene family of this type is at risk of erroneous inferences due to transitive annotation. Consequently, one important prediction of this analysis is that there are uncharacterized functional differences among the several subfamilies.

At the same time, it is clearly possible for an organism to function with a small complement of LHC genes. The chlorophytes *Ostreococcus tauri *and *O. lucimarinus *have highly reduced genomes, and encode clade V LHCs that are similar to other proteins from chlorophytes (including *Chlamydomonas reinhardtii*). The rhodophyte *Cyanidioschyzon merolae*, which also has a highly reduced genome [53], possesses only a single family IIIa LHC as well as two very similar sequences identified by Koziol et al. [2] and additional homologs that did not have the conserved domain architecture or degree of sequence conservation required for inclusion in this study. This is strikingly different from the diversity of genes already identified from EST analyses of red algae. *C. merolae *has been shown to be unusual in other ways, and this is not necessarily surprising [54,55].

### A single subfamily can comprise LHCs that bind different Chl and carotenoids

Clade V comprises LHCs from diatoms, chlorophytes, haptophytes and fucoxanthin-containing dinoflagellates. Chlorophytes possess Chl *a/b *and use lutein as the primary LHC carotenoid, while diatoms, haptophytes and dinoflagellates use Chl *a/c *and fucoxanthin (or derivatives). The grouping of these sequences into a single clade implies that similar LHCs can bind a diverse set of Chls and carotenoids. This observation parallels *in vitro *reconstruction experiments performed by Grabowski et al. [26] where non-native Chl and primary carotenoids were functionally inserted into LhcaR1 from the rhodophyte *P. cruentum *in clade III. The reconstructed LHC was able to functionally bind Chl *a, b *and *c *as well as the carotenoids lutein, fucoxanthin and peridinin in addition to its native zeaxanthin, albeit with some variation in affinity. Thus, it appears that the binding of primary carotenoids and certain chlorophylls is due to the nature of the endogenous biosynthesis of Chlorophyll and carotenoids, and not necessarily because of a profound incongruence in the structure or binding properties of the protein; this inference remains to be tested *in vivo*.

### Functional specialization

Despite the plasticity of pigment binding, the persistence of multiple subfamilies of LHC over hundreds of millions of years of evolutionary divergence (Figure 1) suggest that these subfamilies are functionally distinct. Unfortunately, only a small fraction of the gene products corresponding to the sequences studied here have been characterized biochemically. Nonetheless, placing differences in expression patterns, pigment binding, and differential physical associations among LHCs in a phylogenetic context, it is apparent that individual subfamilies have undergone specialization (See Additional File 3, Supplementary Information).

Plants and chlorophytes have two major types of LHCs, namely LHC I and II, which have distinct biochemistries and associate primarily with PS I and II, respectively. However, the LHCs of rhodophytes and Chl *c*-containing algae constitute a sister clade to most chlorophyte sequences, meaning that they diverged independently of those in the green lineage. Consequently, the diversity of LHCs in Chl *c*-containing algae is generally distinct and independent of that observed in plants and chlorophytes [29], with the notable exception of subfamily Vb. Moreover, the rhodophyte LHCs associate only with PS I, so proteins that associate with PS II like those of chlorophytes are thought to have evolved independently in Chl *c*-containing algae and chlorophytes and generally do not show PS specificity [25]. In rhodophytes an unrelated set of phycobiliproteins are associated with PS II [56].

The sequence diversity and biochemical differences observed in the LHC gene phylogeny suggest that members of the protein family have undergone functional specialization. This complements the work of Koziol et al. [2] on chlorophyte LHCs and extends it to the LHCs of Chl *c*-containing algae. The conservation of LHCs from multiple and diverse subfamilies in individual organisms or closely related lineages suggests that the subfamilies are functionally distinct and could exhibit differences in pigment binding, absorption spectrum, strength of interaction with thylakoid membrane and differences in expression and associations with other LHCs. In addition to the advantages of increased gene dosage, having such a diversity of LHCs may allow an organism to carry out photosynthesis or photoprotection under a range of conditions.

The phylogeny of LHCs observed here cannot be easily reconciled with the currently accepted organismal phylogeny [13], and the presence of many subfamilies that contain proteins from diverse algal lineages is problematic. One explanation would be that the complex phylogeny is the result of extensive horizontal gene transfer between taxa in distant lineages. However, even the most parsimonious scenario would require a very large number of transfer events, and we favor the interpretation that some of the subfamilies are ancestral, and that there is substantial sequence diversity that reflects selection on functionally distinct proteins.

This presents a new perspective on LHC diversity, and illustrates the difficulty inferring functional similarity from sequence similarity. Although there is no doubt that the sequences studied here are all homologous (i.e., are derived from a single common ancestral sequence), the sequence diversity and evolutionary complexity of the gene family are far greater than was appreciated until recently. Most troubling is the biochemical difference between the *Cy. cryptica *Fcp1-3 and Fcp5 in subfamily VIIe2.2 because the former is thought to associate in heterotrimers with Fcp6 and Fcp7 but the latter in higher oligomers despite high sequence similarity. If the relationships shown here are accurate and the biochemical data reliable, then one would predict great potential for functional differences among the many uncharacterized LHCs.

There can be little doubt that a complex history of gene duplication, gene loss, and functional specialization shaped the phylogeny presented here. Closely related paralogs in the same lineage are likely the result of recent gene duplication events, while more distantly related proteins or LHCs that are shared by distant lineages are likely the result of ancestral duplications, or perhaps horizontal gene transfer. Following these ancestral events, gene loss led to the phylogeny observed in this study, with extreme (but not convergent) reduction having occurred in both *Ostreococcus *and *Cyanidioschyzon*. Although much of the apparent gene loss might be attributed to incomplete sampling, the absence of cryptophyte, rhodophyte and peridinin dinoflagellate LHCs from clade V is noteworthy. Proteins from this group are thought to be involved in photoprotection by binding xanthophyll cycle carotenoids and facilitating non-photochemical quenching [22,57]. The presence of a xanthophyll cycle has been demonstrated in all of the lineages with clade V proteins, but to our knowledge has not been demonstrated in cryptophytes or peridinin-containing dinoflagellates. One hypothesis would be that proteins from clade V are involved in the xanthophyll cycle. Brown algae have a xanthophyll cycle, but a brown algal homolog in clade V had not been characterized at the time that the analysis was performed. We speculated that this was a result of the sampling bias described above, and indeed, two very recent studies of brown algae (*Fucus spp*. and *Ectocarpus siliculosus*) found sequences that belong to the clade V LHCs [58,59].

This study characterized the large diversity of LHC sequences and demonstrated that individual species have LHCs from multiple subfamilies, and that subfamilies are not lineage specific. Relating the molecular phylogeny to the biochemistry of the protein has the potential to add substantial insight into the evolution and functional divergence of LHCs. It also tells a cautionary tale for the inference of function solely on the basis of sequence similarity, and suggests the importance of independent lines of evidence in the analysis of genomic data.

## Conclusions

This study provides a comprehensive overview of an important subfamily of LHC genes. Phylogenetic analysis of gene family sequences can provide valuable insights into the diversification of gene families, and a lack of understanding of relationships among LHC sequences has very likely led to incorrect inference of function. Further work will be needed to validate the predictions made here, but it is very likely that several of the clades identified here represent functionally distinct entities, and correctly distinguishing among these sequence variants should help understand their biochemical function. It is also possible that horizontal gene transfer and gene duplication *without *functional differentiation of the duplicated genes have played a role in the evolution of LHCs. Taken together, these observations illustrate the power of comparative sequence analysis for functional inference, but also demonstrate the potential pitfalls in overly simplistic analyses.

## Methods

### Sequence data acquisition

Sequence data from EST surveys of 19 algal species, individual genomic sequences from 25 species, and complete genomic data from 6 species were screened for LHC homologs (Additional File 2, Additional Table S1). Putative LHCs were identified with BLAST [60] using Chl *c*-containing algae LHC sequences from Durnford et al. [29] as queries. The sequences identified by BLAST were clustered with Sequencher (Gene Codes, Ann Arbor, MI, USA) to remove duplicate and nearly identical sequences from the same species. Reads were clustered if they had > 92% identity for a 40 bp overlapping region.

MEME [61] and MAST [62] were used to further characterize the conserved domain structure of the gene family. The Chl *c*-containing algae sequences from Durnford et al. [29] were used as a training dataset to create a local motif profile of the gene family using MEME. MAST was then used to compare the set of BLAST hits against this profile. The characterization of local motifs in the dataset facilitated the identification of a subset of sequences that could be aligned. As expected from previous studies [19,29] the conserved regions corresponded to the three transmembrane regions plus surrounding sequence including Chl and carotenoid binding sites. BLAST hits above an e-value of 1e-19 or without the expected domain structure could not be aligned well with other LHCs and were not included in subsequent analyses. In addition, individual LHCs from polycistronic [63] or polyprotein [64,65] sequences were treated as separate sequences. Sequence data were organized and manipulated using the BioPerl toolkit [66].

### Sequence alignment and phylogenetic analyses

An amino acid alignment of LHC sequences was constructed with ClustalW [67] and was edited manually using MacClade 4.0 (Sinauer Associates, Sunderland, MA, USA). Other publicly available alignment programs were used, but the alignment from ClustalW was deemed to be the most biologically relevant based on the location of gaps in relation to the previously reported conserved regions and the known tertiary structure of the LHC protein. The amino acid alignment was used to create the nucleotide alignment for further analysis [68]. Poorly conserved regions of the protein were omitted from the phylogenetic analysis, so that the alignment consisted mostly of TMRs and each sequence had an average of 149 amino acids out of 266 positions in the alignment. Since these regions span the membrane, the amino acid composition is biased in favor of hydrophobic residues. Because this bias is not adequately addressed by amino acid substitution models and due to the short length of the alignment, both the amino acid and corresponding nucleotide alignment were used for phylogenetic analysis. Preliminary datasets consisted of 470 LHC sequences that fit the criteria above, but individual operational taxonomic units (OTUs) were omitted to reduce the computation time and facilitate the interpretation of the phylogenetic tree. The OTUs corresponding to very similar orthologs within a single lineage were omitted so that the final dataset containing 246 OTUs maintained the same sequence diversity and taxon distribution as the original. Orthologs from complete genomes were not omitted. Comparisons of the topologies from the two datasets indicated that they were consistent. The alignment has been deposited in TreeBase under the accession number 10532.

Phylogenetic analyses based on nucleotides were conducted under the maximum likelihood optimality criterion using the GTR + I + Γ model with 4 rate categories implemented in MrBayes 3.1.2 [39] and Garli 0.942 [38]. The model was selected using MrModeltest 2.2 [69]. MrBayes was run for 10 000 000 generations, trees were sampled every 1000 generations and a burnin of 25% was used to create a consensus tree. Garli was run with the default parameters for the tree search and 200 bootstrap replicates. The same parameters were used to analyze the nucleotide alignment without third codon-position nucleotides.

Phylogenetic analyses of the amino acid alignment were conducted with MrBayes and PhyML 2.4.5 [37]. MrBayes was run with the mixed fixed-rate model and the parameters used above. The consensus tree was used as the starting topology for the PhyML analysis, which used WAG + I + Γ and 8 rate categories for the tree search and 100 bootstrap replicates. The model was selected using ProtTest 1.3 [70]. This tree is presented in Figure 1. See Additional File 1, Additional Figure S1 for equivalent tree with support values from other analyses.

## Authors' contributions

GEH carried out the analysis and drafted the manuscript. MVSP and CFD conceived of the study, and participated in its design and helped to draft the manuscript. All authors read and approved the final manuscript.

## Supplementary Material

Additional file 1 **List of organisms from which LHC sequence data was examined in the current analysis**. Data sources and citation for sequence data use in this analysis Click here for file

Additional File 2 **Detailed LHC phylogeny**. The maximum likelihood tree from PhyML, with 246 taxa based on a 266 aa alignment (PhyML/AA). This tree is equivalent to that in Figure 1, but contains all the sequence names and support values from additional analyses. The support values for each branch where obtained as follows: PhyML with amino acids (top left), MrBayes with amino acids (bottom left), Garli with nucleotides (top right), MrBayes with nucleotides (bottom right). The individual LHCs are colored by taxonomic lineage: chlorophytes (green), cryptophytes (light blue), fucoxanthin-containing dinoflagellates (light purple), peridinin-containing dinoflagellates (dark blue), haptophytes (pink), heterokonts (orange), rhodophytes (red). Click here for file

Additional File 3 **Supplementary Information**. This file contains added information detailing the analytical methods used and details on the interpretation of the tree that are not included in the main manuscript. Click here for file
