# Supplementary material for: Evolution of light-harvesting complex proteins from Chl c-containing algae
Source: BMC Evol Biol. 2011 Apr 15;11:101. doi: 10.1186/1471-2148-11-101 (PMC3096602; doi:10.1186/1471-2148-11-101)
Supplement: Additional file 1 — List of organisms from which LHC sequence data was examined in the current analysis. Data sources and citation for sequence data use in this analysis [file 1471-2148-11-101-S1.PDF]

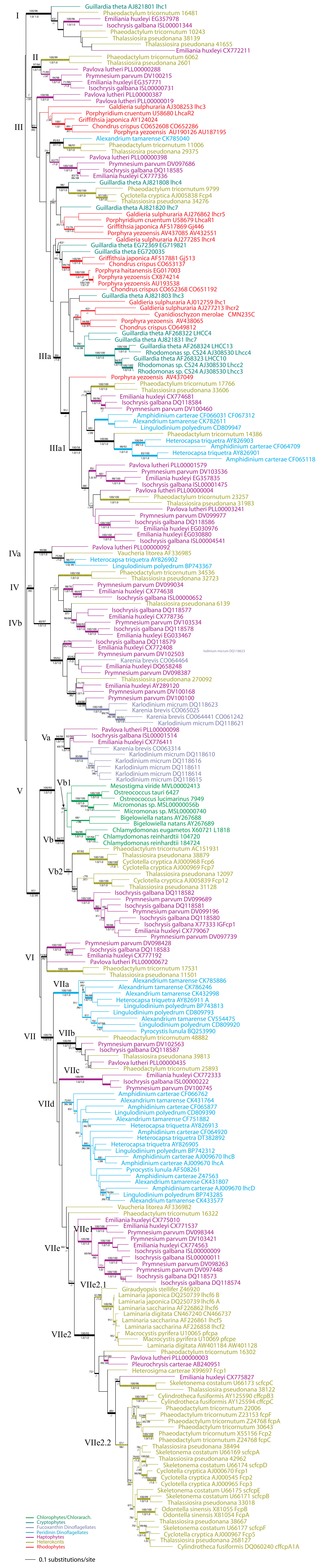

Additional Figure 1:
The maximum likelihood tree from PhyML, with 246 taxa based on a 266 aa alignment (PhyML/AA). This tree is equivalent to that in Figure 2, but contains all the sequence names and support values from additional analyses. The support values for each branch where obtained as follows: PhyML with amino acids (top left), MrBayes with amino acids (bottom left), Garli with nucleotides (top right), MrBayes with nucleotides (bottom right).
