# Supplementary material for: Evolution of light-harvesting complex proteins from Chl c-containing algae
Source: BMC Evol Biol. 2011 Apr 15;11:101. doi: 10.1186/1471-2148-11-101 (PMC3096602; doi:10.1186/1471-2148-11-101)
Supplement: Additional File 2 — Detailed LHC phylogeny. The maximum likelihood tree from PhyML, with 246 taxa based on a 266 aa alignment (PhyML/AA). This tree is equivalent to that in Figure 1, but contains all the sequence names and support values from additional analyses. The support values for each branch where obtained as follows: PhyML with amino acids (top left), MrBayes with amino acids (bottom left), Garli with nucleotides (top right), MrBayes with nucleotides (bottom right). The individual LHCs are colored by taxonomic lineage: chlorophytes (green), cryptophytes (light blue), fucoxanthin-containing dinoflagellates (light purple), peridinin-containing dinoflagellates (dark blue), haptophytes (pink), heterokonts (orange), rhodophytes (red). [file 1471-2148-11-101-S2.DOC]

**Additional** **Table 1:**

List of organisms from which LHC sequence data was examined in the current analysis.

**Additional Table 1**

| **Complete Genomes** | **Source** | **Reference** |
| --- | --- | --- |
| *Chlamydomonas reinhardtii* | JGI^a^ | [1] |
| *Cyanidioschyzon merolae* | *C. merolae* Genome Project^b^ | [2] |
| *Ostreococcus lucimarinus* | JGI | JGI, 2006, version 2.0 |
| *Ostreococcus tauri* | JGI | JGI, 2006, version 2.0 |
| *Phaeodactylum tricornutum* | JGI | JGI ,2006, version 2.0 |
| *Thalassiosira pseudonana* | JGI | Armbrust et al., 2004; JGI, 2006, version 3.0 |
|  |  |  |
| **Expressed sequence tags** | **Source** | **Reference** |
| *Alexandrium tamarense* | GenBank^c^ | [3] |
| *Amphidinium carterae* | GenBank | [4, 5] |
| *Bigelowiella natans*^f^ | TBestDB^d^ | Keeling, 2004, Unpublished |
| *Chondrus crispus* | GenBank | [6] |
| *Emiliania huxleyi* | GenBank | Bonaldo, 2006. Unpublished |
| *Galdieria sulphuraria*^f^ | *G. sulphuraria* Genome Project^e^ | [7] |
| *Guillardia theta* | TBestDB | Keeling, 2006, Unpublished |
| *Heterocapsa triquetra* | GenBank | [8] |
| *Isochrysis galbana* | TBestDB | Keeling, 2006, Unpublished |
| *Karenia brevis* | GenBank | [9] |
| *Karlodinium micrum* | TBestDB | Keeling, 2006, Unpublished |
| *Laminaria digitata* | GenBank | [10] |
| *Lingulodinium polyedrum* | GenBank | [4, 5] |
| *Mesostigma viride* | TBestDB | Lee, 2006, Unpublished |
| *Micromonas* sp. | TBestDB | Durnford, 2006, Unpublished |
| *Pavlova lutheri* | TBestDB | Keeling, 2006, Unpublished |
| *Porphyra haitanensis* | GenBank | [11] |
| *Porphyra yezoensis* | GenBank | Nikaido et al., 2000; Asamizu et al., 2003 |
| *Prymnesium parvum* | GenBank | [12] |

| **Individual Sequences** | **Source** | **Reference** |
| --- | --- | --- |
| *Amphidinium carterae* | GenBank | [13] |
| *Bigelowiella natans* | GenBank | [14] |
| *Chlamydomonas eugametos* | GenBank | [15] |
| *Cyclotella cryptica* | GenBank | [16] |
| *Cylindrotheca fusiformis* | GenBank | [17] |
| *Emiliania huxleyi* | GenBank | [18, 19] |
| *Galdieria sulphuraria* | GenBank | [20, 21] |
| *Giraudyopsis stellifer* | GenBank | [22] |
| *Griffithsia japonica* | GenBank | [23] |
| *Guillardia theta* | GenBank | [24, 25] |
| *Heterosigma carterae* | GenBank | [26] |
| *Isochrysis galbana* | GenBank | La Roche et al., 1994; Patron et al., 2006 |
| *Karlodinium micrum* | GenBank | [27] |
| *Laminaria digitata* | GenBank | [28] |
| *Laminaria japonica* | GenBank | [29] |
| *Laminaria saccharina* | GenBank | [30] |
| *Macrocystis pyrifera* | GenBank | [31] |
| *Odontella sinensis* | GenBank | [32] |
| *Phaeodactylum tricornutum* | GenBank | [33, 34] |
| *Pleurochrysis carterae* | GenBank | [35] |
| *Porphyridium cruentum* | GenBank | [36]; [37] |
| *Pyrocystis lunula* | GenBank | [38] |
| *Rhodomonas sp.* | GenBank | [39] |
| *Skeletonema costatum* | GenBank | [40] |
| *Vaucheria litorea* | GenBank | [41] |

^a^ Joint Genome Institute, http://www.jgi.doe.gov

^b^ *Cyanidioschyzon merolae* Genome Project, http://merolae.biol.s.u-tokyo.ac.jp

^c^ National Center for Biotechnology Information, http://www.ncbi.nlm.nih.gov

^d^ Taxonomically Broad EST Database, http://tbestdb.bcm.umontreal.ca

^e^ *Galdieria sulphuraria* Genome Project, http://genomics.msu.edu/galdieria

^f^ Light harvesting complex proteins identified in these datasets were almost identical to individual genomic sequences from the same organisms, so the EST sequences were not included in the phylogenetic analysis.

**Supplementary Table 1 References:**

1. Merchant SS, Prochnik SE, Vallon O, Harris EH, Karpowicz SJ, Witman GB, Terry A, Salamov A, Fritz-Laylin LK, Maréchal-Drouard L *et al*: **The *Chlamydomonas* genome reveals the evolution of key animal and plant functions**. *Science* 2007, **318**:245-250.

2. Matsuzaki M, Misumi O, Shin-I T, Maruyama S, Takahara M, Miyagishima SY, Mori T, Nishida K, Yagisawa F, Nishida K *et al*: **Genome sequence of the ultrasmall unicellular red alga *Cyanidioschyzon merolae* 10D**. *Nature* 2004, **428**(6983):653-657.

3. Hackett JD, Scheetz TE, Yoon HS, Soares MB, Bonaldo MF, Casavant TL, Bhattacharya D: **Insights into a dinoflagellate genome through expressed sequence tag analysis**. *BMC Genomics* 2005, **6**.

4. Bachvaroff TR, Concepcion GT, Rogers CR, Herman EM, Delwiche CF: **Dinoflagellate expressed indicate massive transfer to the nuclear genome sequence tag data of chloroplast genes**. *Protist* 2004, **155**(1):65-78.

5. Tanikawa N, Akimoto H, Ogoh K, Chun W, Ohmiya Y: **Expressed sequence tag analysis of the dinoflagellate *Lingulodinium polyedrum* during dark phase**. *Photochem Photobiol* 2004, **80**(1):31-35.

6. Collen J, Roeder V, Rousvoal S, Collin O, Kloareg B, Boyen C: **An expressed sequence tag analysis of thallus and regenerating protoplasts of *Chondrus crispus* (Gigartinales, Rhodophyceae)**. *J Phycol* 2006, **42**(1):104-112.

7. Weber APM, Oesterhelt C, Gross W, Brautigam A, Imboden LA, Krassovskaya I, Linka N, Truchina J, Schneidereit J, Voll H *et al*: **EST-analysis of the thermo-acidophilic red microalga *Galdieria sulphuraria* reveals potential for lipid A biosynthesis and unveils the pathway of carbon export from rhodoplasts**. *Plant Mol Biol* 2004, **55**(1):17-32.

8. Patron NJ, Waller RF, Archibald JM, Keeling PJ: **Complex protein targeting to dinoflagellate plastids**. *J Mol Biol* 2005, **348**(4):1015-1024.

9. Lidie KB, Ryan JC, Barbier M, Van Dolah FM: **Gene expression in Florida red tide dinoflagellate *Karenia brevis*: Analysis of an expressed sequence tag library and development of DNA microarray**. *Mar Biotechnol* 2005, **7**(5):481-493.

10. Roeder V, Collen J, Rousvoal S, Corre E, Leblanc C, Boyen C: **Identification of stress gene transcripts in *Laminaria digitata* (Phaeophyceae) protoplast cultures by expressed sequence tag analysis**. *J Phycol* 2005, **41**(6):1227-1235.

11. Fang Y, Fan X, Pang G, Chen B, Wang G, Hu S: **Generation and analysis of 5381 expressed sequence tags (ESTs) from filamentous sporophyte of *Porphyra haitanensis***. *Unpublished* 2006.

12. La Claire JW: **Analysis of expressed sequence tags from the harmful alga, *Prymnesium parvum* (Prymnesiophyceae, Haptophyta)**. *Mar Biotechnol* 2006, **8**(5):534-546.

13. Hiller RG, Wrench PM, Sharples FP: **The light-harvesting chlorophyll a\c-binding protein of dinoflagellates - a putative polyprotein**. *FEBS Lett* 1995, **363**(1-2):175-178.

14. Archibald JM, Rogers MB, Toop M, Ishida K, Keeling PJ: **Lateral gene transfer and the evolution of plastid-targeted proteins in the secondary plastid-containing alga *Bigelowiella natans***. *Proc Natl Acad Sci USA* 2003, **100**(13):7678-7683.

15. Gagne G, Guertin M: **The early genetic response to light in the green unicellular alga *Chlamydomonas eugametos* grown under light dark cycles involves genes that represent direct responses to light and photosynthesis**. *Plant Mol Biol* 1992, **18**(3):429-445.

16. Eppard M, Krumbein WE, von Haeseler A, Rhiel E: **Characterization of fcp4 and fcp12, two additional genes encoding light harvesting proteins of *Cyclotella cryptica* (Bacillariophyceae) and phylogenetic analysis of this complex gene family**. *Plant Biol* 2000, **2**(3):283-289.

17. Poulsen N, Kroger N: **A new molecular tool for transgenic diatoms - Control of mRNA and protein biosynthesis by an inducible promoter-terminator cassette**. *FEBS J* 2005, **272**(13):3413-3423.

18. Corstjens PL, Gonzalez EL: **Effects of nutrient limitation and stress on the expression of the coccolith-vesicle V-ATPase (subunit c) of *Pleurochrysis***. *Unpublished* 2003.

19. Quinn P, Bowers RM, Zhang YY, Wahlund TM, Fanelli MA, Olszova D, Read BA: **cDNA microarrays as a tool for identification of biomineralization proteins in the coccolithophorid *Emiliania huxleyi* (Haptophyta)**. *Appl Environ Microbiol* 2006, **72**(8):5512-5526.

20. Marquardt J, Wans S, Rhiel E, Randolf A, Krumbein WE: **Intron-exon structure and gene copy number of a gene encoding for a membrane-intrinsic light-harvesting polypeptide of the red alga *Galdieria sulphuraria***. *Gene* 2000, **255**(2):257-265.

21. Marquardt J, Rhiel E: **Genomic sequences for light-harvesting proteins of photosystem I of the red alga *Galdieria sulphuraria***. *Unpublished* 2006.

22. Passaquet C, Lichtl C: **Molecular Study of a Light-Harvesting Apoprotein of *Giraudyopsis Stellifer* (Chrysophyceae)**. *Plant Mol Biol* 1995, **29**(1):135-148.

23. Liu CL, Huang XH, Lee Y, Lee H, Li GY: **Characteristics and phylogeny of light-harvesting complex gene encoded proteins from marine red alga *Griffithsia japonica***. *Acta Oceanologica Sinica* 2005, **24**(2):120-130.

24. Gould SB, Sommer MS, Hadfi K, Zauner S, Kroth PG, Maier UG: **Protein targeting into the complex plastid of cryptophytes**. *J Mol Evol* 2006, **62**(6):674-681.

25. Deane JA, Fraunholz M, Su V, Maier UG, Martin W, Durnford DG, McFadden GI: **Evidence for nucleomorph to host nucleus gene transfer: Light-harvesting complex proteins from cryptomonads and chlorarachniophytes**. *Protist* 2000, **151**(3):239-252.

26. Durnford DG, Aebersold R, Green BR: **The fucoxanthin-chlorophyll proteins from a chromophyte alga are part of a large multigene family: Structural and evolutionary relationships to other light harvesting antennae**. *Mol Gen Genet* 1996, **253**(3):377-386.

27. Patron NJ, Waller RF, Keeling PJ: **A tertiary plastid uses genes from two endosymbionts**. *J Mol Biol* 2006, **357**(5):1373-1382.

28. Crepineau F, Roscoe T, Kaas R, Kloareg B, Boyen C: **Characterisation of complementary DNAs from the expressed sequence tag analysis of life cycle stages of *Laminaria digitata* (Phaeophyceae)**. *Plant Mol Biol* 2000, **43**(4):503-513.

29. Zhou Z-G, Bi Y-H, Shi X-Z: **Isolation and characterization of a differentially expressed gene, lhcf6, encoding light-harvesting fucoxanthin-chlorophyll c antenna protein from male gametophyte of *Laminaria japonica***. *Unpublished* 2006.

30. De Martino A, Douady D, Quinet-Szely M, Rousseau B, Crepineau F, Apt K, Caron L: **The light-harvesting antenna of brown algae - Highly homologous proteins encoded by a multigene family**. *Eur J Biochem* 2000, **267**(17):5540-5549.

31. Apt KE, Clendennen SK, Powers DA, Grossman AR: **The gene family encoding the fucoxanthin chlorophyll proteins from the brown alga *Macrocystis pyrifera***. *Mol Gen Genet* 1995, **246**(4):455-464.

32. Kroth-Pancic PG: **Nucleotide sequence of 2 cDNAs encoding fucoxanthin chlorophyll a/c proteins in the diatom *Odontella sinensis***. *Plant Mol Biol* 1995, **27**(4):825-828.

33. Grossman A, Manodori A, Snyder D: **Light-harvesting proteins of diatoms - Their relationship to the chlorophyll a/b binding-proteins of higher-plants and their mode of transport into plastids**. *Mol Gen Genet* 1990, **224**(1):91-100.

34. Grossman AR, Schaefer MR, Chiang GG, Collier JL: **The phycobilisome, a light-harvesting complex responsive to environmental conditions**. *Microbiol Rev* 1993, **57**(3):725-749.

35. Sakurai T, Hwang S, Tohse H, Nagasawa H: **Differentially expressed genes of *Pleurochrysis carterae***. *Unpublished* 2006.

36. Tan S, Cunningham FX, Gantt E: **LhcaR1 of the red alga *Porphyridium cruentum* encodes a polypeptide of the LHCI complex with seven potential chlorophyll a-binding residues that are conserved in most LHCs**. *Plant Mol Biol* 1997, **33**(1):157-167.

37. Tan S, Ducret A, Aebersold R, Gantt E: **Red algal LHC I genes have similarities with both Chl a/b- and a/c-binding proteins: A 21 kDa polypeptide encoded by LhcaR2 is one of the six LHC I polypeptides**. *Photosynthesis Res* 1997, **53**(2-3):129-140.

38. Okamoto OK, Hastings JW: **Novel dinoflagellate clock-related genes identified through microarray analysis**. *J Phycol* 2003, **39**(3):519-526.

39. Broughton MJ, Howe CJ, Hiller RG: **Distinctive organization of genes for light-harvesting proteins in the cryptophyte alga *Rhodomonas***. *Gene* 2006, **369**:72-79.

40. Smith GJ, Gao Y, Alberte RS: **The fucoxanthin-chlorophyll a/c proteins comprise a large family of coexpressed genes in the marine diatom *Skeletonema costatum* (Greve)**. *Plant Physiol* 1997, **114**:1136.

41. Summer EJ, Rumpho ME: **Chloroplast localized, nuclear encoded proteins persist for many months in an animal cell despite the lack of cognate algal nuclear genes**. *Unpublished* 2001.
