# Supplementary material for: Evolution of light-harvesting complex proteins from Chl c-containing algae
Source: BMC Evol Biol. 2011 Apr 15;11:101. doi: 10.1186/1471-2148-11-101 (PMC3096602; doi:10.1186/1471-2148-11-101)
Supplement: Additional File 3 — Supplementary Information. This file contains added information detailing the analytical methods used and details on the interpretation of the tree that are not included in the main manuscript. [file 1471-2148-11-101-S3.DOCX]

**Supplementary Information**

*Detailed discussion of the phylogeny*

Experimental data are lacking from LHCs in clades I and II, which contain sequences from three and two lineages, respectively. Clade III contains a wide diversity of LHCs from five lineages distributed in subfamilies. Many of the deep branches in this clade are poorly supported (and consequently are not individually named in Figure 1), although rhodophyte and cryptophyte LHCs tend to be closely related, while LHCs from haptophytes, diatoms (heterokonts) and peridinin-containing dinoflagellates usually group together in subfamilies (i.e. IIIa1). Clade IV includes four lineages including fucoxanthin-containing dinoflagellates, which are embedded in haptophyte subfamilies. This association has not been characterized previously but is not surprising, considering that this lineage of dinoflagellates has a plastid of haptophyte origin [1] and gene transfer from the haptophyte endosymbiont to the dinoflagellate host has been inferred previously [2]. The only LHCs from clades I-III that have been studied biochemically are LhcaR1 and LhcaR2 from *Porphyridium cruentum*, and Fcp4 from the *Cyclotella cryptica*, which are in clade III, while members of clade IVb were only recently studied in *Emiliania huxleyi* [3]. The relationship among clades I, II, III, and IV has poor bootstrap support and is dependent upon the analytical method.

Clades V, VI and VII form a well-supported cluster. Clade V contains LHCs from an assemblage of lineages that includes haptophytes, diatoms, fucoxanthin-containing dinoflagellates, and strikingly, the *Chl a*/*b* pigmented chlorophytes and chlorarachniophytes.  *Cy. cryptica* Fcp6, Fcp7, Fcp12, *Chlamydomonas eugametos* LI818 and *Ch. reinhardtii* LI818r-1 from this group have been characterized experimentally and are thought to be involved in photoprotection from excess light [4, 5]. Despite the distant organismal relationship between chlorophytes and the other three lineages, the LHCs in clade V are clearly closely related, as characterized previously by Eppard et al. [4] and Richard et al. [5]. Similar to clade IV, sequences from fucoxanthin-containing dinoflagellates in clade V are embedded in a group of haptophytes (i.e. Va). In subfamily Vb, LHCs from haptophytes and chlorophytes are each monophyletic, while those from diatoms are not.

Clade VI is a small clade composed of sequences from heterokonts and haptophytes, and is essentially unstudied, while clade VII is large and contains most of the sequences that have been included in previous analyses. Peridinin-containing dinoflagellates have LHCs from two monophyletic groups within clade VII (VIIa and VIId), while haptophytes have members from three (VIIc, VIIe1 and VIIe2). Subfamily VIIe2 contains the sequences that are thought to be the primary photosynthetic LHCs in heterokonts. Most of the previously studied Chl c-containing algal LHCs are in this subfamily and include *Cy. cryptica* Fcp1, Fcp2, Fcp3, Fcp5, *Macrocystis pyrifera* pfcpa, and those from *P. tricornutum*. These LHCs have been studied experimentally, but we are unfamiliar with any such information for those of *Cylindrotheca* *fusiformis*, *Skeletonema* *costatum*, or *Heterosigma* *carterae*. In addition, there are some spectral and fractionation data on LHCs from *Laminaria* spp., but the data have not been associated with individual genes [6, 7]. The only LHCs from brown algae (heterokonts) included in this analysis are located in clade VIIe2.1. In addition, subfamily VIIe2 included three LHCs from haptophytes. The relationships between the subfamilies of clade VII are unresolved and are not well-supported in other analyses. It is also notable that the monophyly of subfamily VIId is not supported when third codon positions are excluded from the analysis.

*Functional Specialization*

*Ch. reinhardtii* LI818r-1, *Ch. eugametos* LI818 and *Cy. cryptica* Fcp6 and Fcp7 in clade V have been shown to have different expression patterns than *Cy. cryptica* Fcp1-3 and 5 in subfamily VIIe2.2. The *Ch. reinhardtii* and *Ch. eugametos* proteins from clade V are expressed immediately after exposure to light, and expression reaches a maximum within 1 hr. In contrast, the chlorophyte LHC I and II, whose role in photosynthesis is relatively well characterized (but which belong to a different LHC subfamily than that studied here), show a delay in initiation of expression and peak after 4 to 8 hr [8, 9]. In addition, *Ch. eugametos* LI818 in clade V has been shown to be under circadian control and was expressed in anticipation of light stimulus [8]. Moreover, Savard et al. [9] and Richard et al. [5] observed that both LI818 and LI818r-1 were maximally expressed under high light conditions, while expression under low light was minimal. Lefebvre et al. [3] report similar results concerning clade V genes in *E. huxleyi*. Moreover, a similar pattern was observed for *Cy. cryptica* Fcp6 and Fcp7 in clade V and affected mRNA expression, protein levels, and the number of LHCs present in the thylakoid membrane [10-14]. In contrast, these experiments indicated that *Cy. cryptica* Fcp1-3 and 5 from subfamily VIIe2.2 exhibited a delay in expression, and were maximally expressed under low light conditions.

Proteins from clade V and subfamily VIIe2.2 also exhibit specificity in their association with other LHCs to form antenna complexes of trimers or higher oligomers. Studies on *Cy. meneghiniana,* whose LHCs are similar to those of *Cy. cryptica,* indicate that heterotrimers consist of Fcp6 and Fcp7 from clade V and Fcp1-3 from subfamily VIIe2.2 that associate together in a nonstoichiometric ratio [15, 16]. Under high light conditions, the proportion of clade V proteins present in the trimers increases, as is consistent with the previous experimental data. Higher oligomers of LHCs consisted primarily of Fcp5 from subfamily VIIe2.2 [15].

Moreover, differences in pigment types and levels have been observed for each major group. As mentioned above, LHCs from clade V are present in a diverse set of lineages, which utilize different Chl and carotenoid molecules. It has also been demonstrated that more fucoxanthin is present in *Cy. cryptica* LHCs isolated under low light than high light conditions, while levels of diadinoxanthin and diatoxanthin, both xanthophyll cycle carotenoids, increases under high light [12, 17]. Beer et al. [15] observed that the increase in clade V proteins in the heterotrimeric LHCs under high light was accompanied by an increase in diadinoxanthin and diatoxanthin levels, while Guglielmi et al. [18] made similar observations in *P. tricornutum*. The correlation between the increase in Fcp6 and Fcp7 and the change in carotenoid composition led Beer et al. [15] to conclude that these clade V LHCs bind xanthophyll cycle carotenoids, while clade VIIe2.2 LHCs bind fucoxanthin more exclusively.

The observation of the expression patterns and pigment binding of clade V LHCs has suggested that these proteins play a role in photoprotection, while subfamily VIIe2.2 proteins form the major antennas for photosynthesis [9, 15]. Under low light conditions, clade VII LHCs are maximally expressed and fucoxanthin levels are highest in order to absorb the maximum amount of light for photosynthesis. Under high light conditions, excess light can damage PS II, lead to photo-oxidation and the creation of oxygen radicals (Muller et al. 2001). Therefore, the continued absorption of photons and transfer of energy to the reaction center can be deleterious. Some carotenoids have the ability to decouple photon absorption and energy transfer to PS II by dissipating excess energy via nonphotochemical quenching (NPQ) (Muller et al. 2001). This process involves the xanthophyll cycle whereby carotenoids are de-epoxidized to dissipate the energy. In diatoms, this cycle includes diadinoxanthin and diatoxanthin, but other algae use different carotenoids. Because of the expression pattern and the fact that clade V LHCs preferentially associate with xanthophyll cycle carotenoids, we speculate that the LHCs from this clade are primarily involved in the xanthophyll cycle or NPQ. Since NPQ diverts energy that would otherwise be used in photosynthesis, the presence of photoprotective LHCs under low light conditions could be disadvantageous. Thus the ratio of clade V to subfamily VIIe2.2 proteins present in trimers decreases under low light. Indeed, Beer et al. [15] observe increased NPQ with an increase in Fcp6, Fcp7 and xanthophyll cycle carotenoids.

The division of LHC proteins into functionally distinct and evolutionarily related clades has also been observed by Koziol et al. [19]. That analysis focused on chlorophyte and plant LHCs and concluded that diversity in those lineages is separate from the diversity observed in the current study, with the notable exception of the LI818 family (clade V). The current study focuses on Chl c containing algal LHCs, so most chlorophyte LHCs and other proteins in the superfamily were too distantly related to be included in the analysis. This study identifies many novel subfamilies that are separate from the diversity observed in chlorophyte LHCs, but there are substantially fewer experimental data relating to the proteins included in the current analysis. Therefore, little is known about the specific biochemistry of the individual LHCs and the functions attributed to each subfamily are speculative.

The analysis of Koziol et al. [19] includes LI818-like proteins (clade V), the major Chl c-containing algal LHCs (clade VII), rhodophyte and cryptophyte LHCs (clade III) and the novel Lhcz protein (clade I). However, the current analysis includes more sequences from a greater number of lineages so that the sequence diversity in individual lineages can be examined. In addition, the current analysis identifies 3 novel clades and many more novel subfamilies. Since the current analysis did not include the chlorophyte Lhca and Lhcb families, the relationships among Chl c-containing algal LHCs were better supported. Specifically, the LI818 proteins (clade V) shows a strong association with clade VII, while Koziol et al. [19] found the groups as sister to both major group of Chl c-containing algal LHCs and chlorophyte Lhca and Lhcb. However, based on our phylogeny showing a strong association between clades V and VII, Lhca and Lhcb sequences form a monophyletic outgroup to the sequences analyzed here, but the root of the phylogeny could not be confidently inferred.

The LHC phylogeny of Koziol et al. [19] correlated well with the organismal phylogeny, so the presence or absence of subfamilies could be used to construct an evolutionary history of gene gain and loss in each lineage. The LI818 subfamily (clade V) was, as in this study, observed in both chlorophyte and Chl c-containing lineages and it was concluded that the subfamily emerged very early in algal evolution. Finally, it should be noted that the association of sequences from *Bigelowiella natans* with Lhcz and the major Chl c-containing clade in Koziol et al. [19], were not well supported in that analysis and were omitted from this analysis due to poor alignment.

*Future work*

Further work must be done to experimentally characterize novel LHC subfamilies and determine to what degree the function of closely related LHCs is conserved between phylogenetically diverse species. *P. tricornutum* can serve as a model to test whether the expression patterns and biochemistry of *Cyclotella* spp. LHCs are conserved at least within diatoms. Biochemical experiments in *P. tricornutum* have so far shown similar increases in xanthophyll cycle carotenoids under high light conditions [20] and similar trimer- and oligomerization of LHCs [21], although the protein composition in both arrangements are thought to be almost identical. In addition, Lepetit [21] and Gugleilmi [18] have observed minor FCPs that do not correspond to the major diatom LHC clade VIIe2 and which likely correspond to one of the other clades described here. In all of these cases, more work needs to be done to associate the proteins with individual genes in the LHC family. Overall, most studies have focused on clade VIIe and have not addressed the putative photoprotective proteins of clade V. There are few data about LHCs from clade III and currently no experimental data concerning the clades I, II, IV, or VI or proteins from fucoxanthin-containing dinoflagellates in clades IV and V.

**References**

1. Tengs T, Dahlberg OJ, Shalchian-Tabrizi K, Klaveness D, Rudi K, Delwiche CF, Jakobsen KS: **Phylogenetic analyses indicate that the 19 ' hexanoyloxy-fucoxanthin-containing dinoflagellates have tertiary plastids of haptophyte origin**. *Mol Biol Evol* 2000, **17**(5):718-729.

2. Ishida K, Green BR: **Second- and third-hand chloroplasts in dinoflagellates: Phylogeny of oxygen-evolving enhancer 1 (PsbO) protein reveals replacement of a nuclear-encoded plastid gene by that of a haptophyte tertiary endosymbiont**. *Proc Natl Acad Sci USA* 2002, **99**(14):9294-9299.

3. Lefebvre SC, Gayle H, Richard W, Nikos L, Richard JG, Christine AR, Betsy AR, Jose LG: **Characterization and expression analysis of the lhcf gene family in Emiliania huxleyi (haptophyta) reveals differential responses to light and CO2**. *J Phycol* 2010, **46**(1):123-134.

4. Eppard M, Rhiel E: **Investigations on gene copy number, introns and chromosomal arrangement of genes encoding the fucoxanthin chlorophyll a/c-binding proteins of the centric diatom *Cyclotella cryptica***. *Protist* 2000, **151**(1):27-39.

5. Richard C, Ouellet H, Guertin M: **Characterization of the LI818 polypeptide from the green unicellular alga *Chlamydomonas reinhardtii***. *Plant Mol Biol* 2000, **42**(2):303-316.

6. Pascal AA, Caron L, Rousseau B, Lapouge K, Duval JC, Robert B: **Resonance Raman spectroscopy of a light-harvesting protein from the brown alga *Laminaria saccharina***. *Biochemistry* 1998, **37**(8):2450-2457.

7. De Martino A, Douady D, Quinet-Szely M, Rousseau B, Crepineau F, Apt K, Caron L: **The light-harvesting antenna of brown algae - Highly homologous proteins encoded by a multigene family**. *Eur J Biochem* 2000, **267**(17):5540-5549.

8. Gagne G, Guertin M: **The early genetic response to light in the green unicellular alga *Chlamydomonas eugametos* grown under light dark cycles involves genes that represent direct responses to light and photosynthesis**. *Plant Mol Biol* 1992, **18**(3):429-445.

9. Savard F, Richard C, Guertin M: **The *Chlamydomonas reinhardtii* LI818 gene represents a distant relative of the cabI/II genes that is regulated during the cell cycle and in response to illumination**. *Plant Mol Biol* 1996, **32**(3):461-473.

10. Becker F, Rhiel E: **Immuno-electron microscopic quantification of the fucoxanthin chlorophyll a/c binding polypeptides Fcp2, Fcp4, and Fcp6 of *Cyclotella cryptica* grown under low- and high-light intensities**. *Int Microbiol* 2006, **9**(1):29-36.

11. Janssen M, Bathke L, Marquardt J, Krumbein WE, Rhiel E: **Changes in the hot apparatus of diatoms in response to low and high light intensities**. *Int Microbiol* 2001, **4**:27-33.

12. Oeltjen A, Krumbein WE, Rhiel E: **Investigations on transcript sizes, steady state mRNA concentrations and diurnal expression of genes encoding fucoxanthin chlorophyll a/c light harvesting polypeptides in the centric diatom *Cyclotella cryptica***. *Plant Biol* 2002, **4**(2):250-257.

13. Oeltjen A, Marquardt J, Rhiel E: **Differential circadian expression of genes fcp2 and fcp6 in *Cyclotella cryptica***. *Int Microbiol* 2004, **7**(2):127-131.

14. Westermann M, Rhiel E: **Localisation of fucoxanthin chlorophyll a/c-binding polypeptides of the centric diatom *Cyclotella cryptica* by immuno-electron microscopy**. *Protoplasma* 2005, **225**(3-4):217-223.

15. Beer A, Gundermann K, Beckmann J, Buchel C: **Subunit composition and pigmentation of fucoxanthin-chlorophyll proteins in diatoms: Evidence for a subunit involved in diadinoxanthin and diatoxanthin binding**. *Biochemistry* 2006, **45**(43):13046-13053.

16. Buchel C: **Fucoxanthin-chlorophyll proteins in diatoms: 18 and 19 kDa subunits assemble into different oligomeric states**. *Biochemistry* 2003, **42**(44):13027-13034.

17. Rhiel E, Marquardt J, Eppard M, Morschel E, Krumbein WE: **The light harvesting system of the diatom *Cyclotella cryptica*. Isolation and characterization of the main light harvesting complex and evidence for the existence of minor pigment proteins**. *Botanica Acta* 1997, **110**(2):109-117.

18. Guglielmi G, Lavaud J, Rousseau B, Etienne AL, Houmard J, Ruban AV: **The light-harvesting antenna of the diatom *Phaeodactylum tricornutum* - Evidence for a diadinoxanthin-binding subcomplex**. *FEBS J* 2005, **272**(17):4339-4348.

19. Koziol A, Borza T, Ishida K-I, Keeling P, Lee RW, Durnford DG: **Tracing the evolution of the light-harvesting antennae in chlorophyll a/b-containing organisms**. *Plant Physiol* 2007, **143**:1802-1816.

20. Schumann A, Goss R, Jakob T, Wilhelm C: **Investigation of the quenching efficiency of diatoxanthin in cells of *Phaeodactylum tricornutum* (Bacillariophyceae) with different pool sizes of xanthophyll cycle pigments**. *Phycologia* 2007, **46**(1):113-117.

21. Lepetit B, Volke D, Szabo M, Hoffmann R, Garab GZ, Wilhelm C, Goss R: **Spectroscopic and molecular characterization of the oligomeric antenna of the diatom Phaeodactylum tricornutum**. *Biochemistry* 2007, **46**(34):9813-9822.
